# Radiation-induced SOD2 overexpression sensitizes colorectal cancer to radiation while protecting normal tissue

**DOI:** 10.18632/oncotarget.13954

**Published:** 2016-12-15

**Authors:** Zhiqiang Zhang, Jinyi Lang, Zhi Cao, Rong Li, Xingyong Wang, Weidong Wang

**Affiliations:** ^1^ Department of Critical Care Medicine, Children's Hospital of Chongqing Medical University, Chongqing 400014, China; ^2^ Department of Radiation Oncology, Sichuan Cancer Hospital, Chengdu 610041, China; ^3^ Department of Radiation Oncology, Sichuan Oncology Institute of Guangxi Medical University, Chengdu 610041, China; ^4^ Institute of Combined Injuries, College of Military Preventive Medicine, Third Military Medical University, Chongqing 400038, China

**Keywords:** superoxide dismutase 2, colorectal cancer, early growth response gene-1, promoter, radiation

## Abstract

This study investigated whether radiation-induced overexpression of superoxide dismutase 2 (SOD2) exerts radio-sensitizing effects on tumor cells while having radio-protective effects on normal cells during radio-activated gene therapy for human colorectal cancer. A chimeric promoter, C_9_BC, was generated by directly linking nine tandem CArG boxes to a CMV basic promoter, after which lentiviral vectors containing GFP and SOD2 gene driven by the C_9_BC promoter were constructed. Stably transfected HT-29 colorectal cancer cells and CCD 841 CoN normal colorectal cells were irradiated to a dose of 6-Gy, and cell proliferation and apoptosis were observed. Tumor xenografts and peritumoral skin tissue in BALB/c mice were infected with the therapeutic lentivirus and subsequently irradiated with a total dose of 6 Gy. *In vitro* experiments revealed that radiation-induced SOD2 overexpression inhibited tumor cell proliferation (61.89% vs. 40.17%, *P* < 0.01) and decreased apoptosis among normal cells (14.8% vs. 9.6%, *P* = 0.02) as compared to untransfected cells. Similar effects were observed *in vivo*. Thus radiation-induced SOD2 overexpression via the chimeric C_9_BC promoter increased the radiosensitivity of HT-29 human colorectal cancer cells and concurrently protected normal CCD 841 CoN colorectal cells from radiation damage.

## INTRODUCTION

Colorectal cancer is the third most common cancer diagnosed in both men and women in the United States [1], and accounts for a fifth of the incidences of cancer in China [2]. Radiotherapy is one of the key countermeasures required for comprehensive therapy of colorectal cancer [3]. However, in the local treatment of a tumor, radiotherapy might damage normal tissue around the tumor [4, 5]. In clinical practice, radiation dose or duration is controlled to mitigate the radiation injury to normal tissue, which leads to decreased efficacy of radiotherapy [6]. Some methods, such as multileaf collimator, precise fixation technique, and radioprotectors have been used to eliminate radiation injury, but with unsatisfactory results. Radioprotectors failed to improve the therapeutic gain ratio of radiotherapy because they protect the normal tissue and tumor cells equally [7, 8]. Radiosensitizers can enhance the tumor radiosensitivity, but might concurrently radiosensitize the normal tissue, resulting in unintended radiation injury.

Recently, gene therapy has been used to treat cancer by activating suppressor genes, suppressing oncogenes, importing cytotoxic genes, improving tumor immunogenicity, and changing the tumor microenvironment. Superoxide dismutase 2 (SOD2) is located in the mitochondrial matrix and serves as an effective radioprotector by clearing cellular oxygen radicals and thus alleviating oxidative damage [9, 10]. SOD2 inhibits tumor proliferation and metastasis and improves the radiosensitivity of tumor cells [11, 12]. Unfortunately, the lack of effective methods for gene transfection and the limited regulation of gene expression in radiation fields limit the clinical application of SOD2 gene therapy .

The promoter region of gene Egr-1 contains six CArG boxes, and this region has a radiation-inducible regulatory sequence that responds directly to radiation [13, 14]. Construction of a chimeric promoter containing a CArG box can optimize the driven efficiency of the promoter and decrease the normal background expression. Additionally, construction of a radiation-inducible chimeric promoter with which SOD2 couples downstream may precisely regulate SOD2 gene expression through controlling radiation dose and duration, and ultimately result in the spatio-temporal restrictive expression of SOD2.

To solve the problem of increased tumor radiosensitivity aggravating normal-tissue damage, we attempted to construct the SOD2 overexpressing lentivirus vector that is driven by a radiation-inducible chimeric promoter. By utilizing this kind of lentivirus vector and controlling the radiation dose and duration, we demonstrated targeted and regulated gene therapy Furthermore, we increased the sensitivity of tumor radiotherapy as well as decreased the radiation-induced normal-tissue damage during radiation treatment. Radiation-induced overexpression of SOD2 can increase the efficacy of tumor radiotherapy and improve the quality of life of cancer patients. Therefore, gene therapy targeting SOD2 has potential in cancer radiotherapy.

## RESULTS

### Radiation-induced GFP expression under different radiation doses

**Figure 1:**
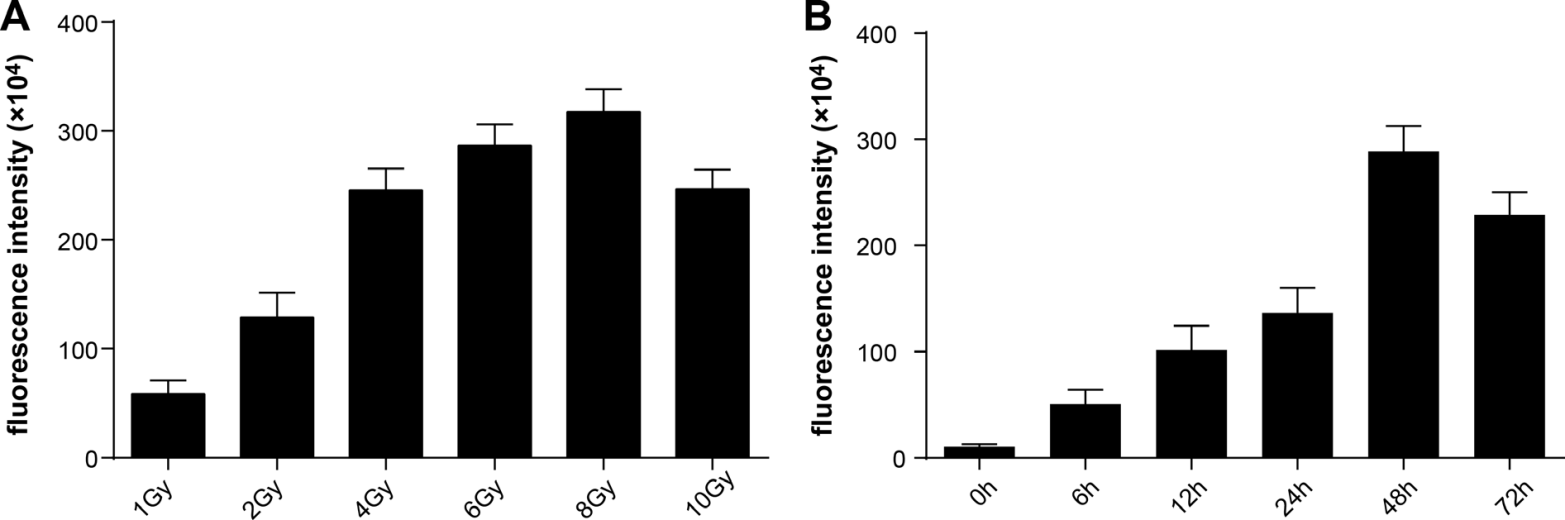
Analysis of radiation-induced features of the reporter vector GFP fluorescence intensity was assessed by flow cytometry. (**A**) The variations of fluorescence intensity at 24 hours after receiving different doses of radiation. (**B**)The variations of fluorescence intensity at different times after receiving 2-Gy doses of radiation.

To choose the appropriate radiation dose and to evaluate the radiation-inducible transcriptional activity of the chimeric promoter, we detected GFP fluorescence intensity of HT-29 cells receiving different radiation doses by flow cytometry. HT-29 cells that stably expressed pLVX-C_9_BC-AcGFP1-N1 were given 1- to 10-Gy doses of g ray radiation, and GFP fluorescence intensity was measured after 24 hours of radiation treatment. Figure 1A shows that the radiation dose of 1 to 8 Gy can effectively induce the expression of GFP genes, and the GFP expression gradually increased with the increase of the radiation dose. A dose larger than 8 Gy decreases fluorescence intensity, which might be to the result of a large dose of radiation causing damage to cells and disrupting cell function.

### Dynamic changes in GFP expression after 2-Gy irradiation of cultured cells

As the conventional dose fractionation in therapy, 2 Gy was used to irradiate the cells. After treatment, GFP expression level in the cells was increased, with increments of 3.8-fold, 8.5-fold, and 11.8-fold at 6 hours, 12 hours, and 24 hours after radiation, respectively, compared with the preradiation baseline. The GFP expression increased to the peak level at 48 hours after radiation (26.2-fold), and significantly decreased after 72 hours (20.5-fold) (Figure 1B).

### SOD2 expression at different times under a 2-Gy radiation dose

We took 2 Gy as the experimental radiation dose to evaluate SOD2 expression in HT-29 cells that stably expressed pLVX-C9BC-SOD2-T2A-AcGFP1. After radiation treatment, SOD2 expression was increased by 1.1-fold, 1.8-fold, and 2.2-fold in 6 hours, 12 hours, and 24 hours after radiation compared with baseline, respectively, with a peak level 48 hours after radiation (2.8-fold), and significantly decreased 72 hours (1.9-fold) after radiation. (Supplementary Figure S1A, S1B). The results indicate that the chimeric promoter can effectively activate the expression of SOD2 when 2-Gy doses of radiation are applied, and the SOD2 expression was most prominent in 24 to 48 hours.

### SOD2 expression in stably transfected cells after radiation treatment

**Figure 2:**
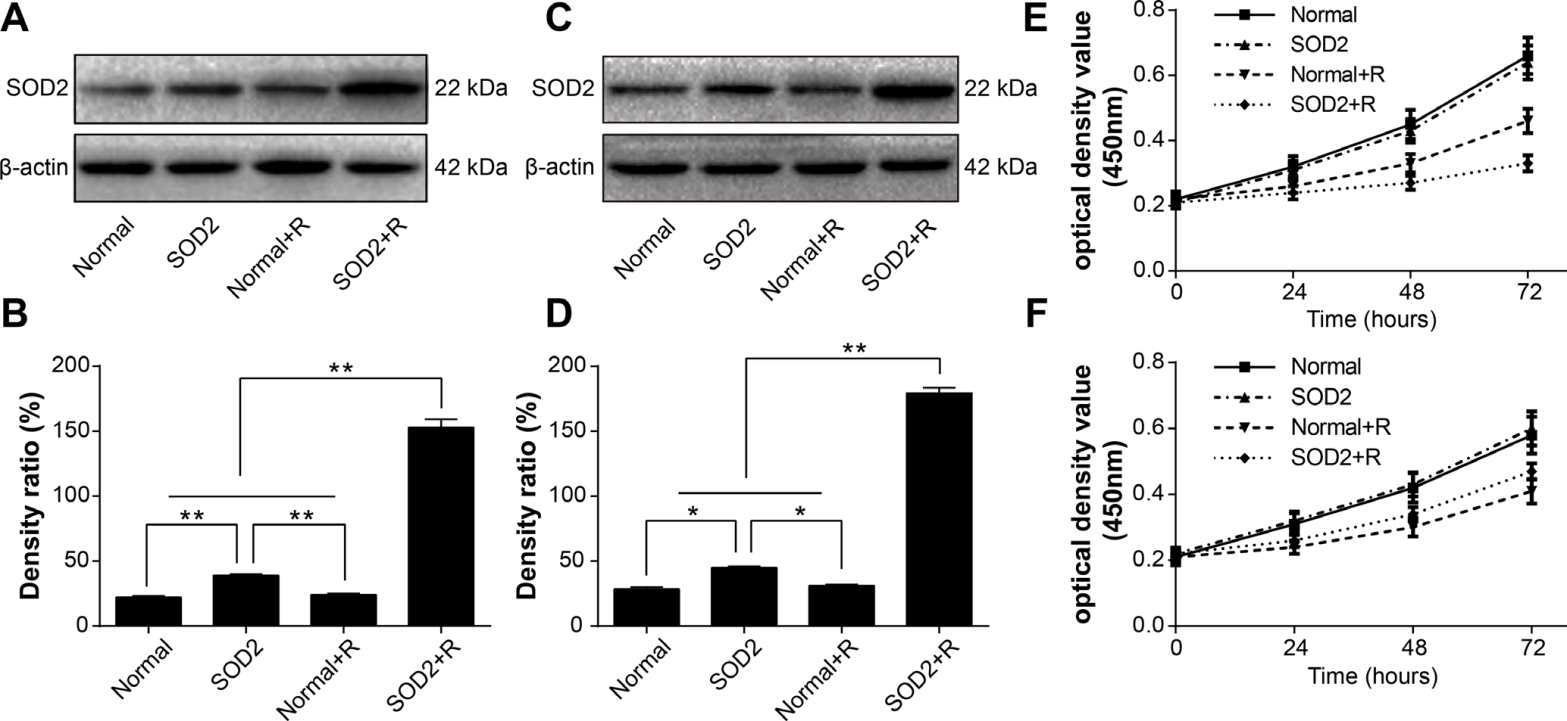
The variations of SOD2 expression and cell proliferation in cells receiving different treatments (**A**) Western blot analysis of SOD2 expression levels in HT-29 cell lines. (**B**) Quantification of SOD2 expression by Western blot in HT-29 cell lines. (**C**) Western blot analysis of SOD2 expression levels in CCD 841 CoN cell lines. (**D**) Quantification of SOD2 expression by Western blot in CCD 841 CoN cell lines. (**E**) HT-29 cell proliferation was measured with Cell Counting Kit-8 assay. (**F**) CCD 841 CoN cell proliferation was measured with Cell Counting Kit-8 assay. Data are shown as the means ± SD from three independent experiments. *P < 0.05; ^*^P < 0.01. Normal: cells without treatment. SOD2: cells stably expressed SOD2 therapeutic vector. Normal + R: cells receiving 6 Gy of radiation treatment.SOD2 + R: cells stably expressed SOD2 therapeutic vector receiving 6 Gy of radiation treatment.

SOD2 expression can be activated by radiation doses of 2 to 8 Gy. Considering our previous findings along with conventional fractionated radiotherapy, we used a total radiation dose of 6 Gy in both *in vivo* and *in vitro* experiments. *In vivo* experiments utilized a 2-Gy dose of radiation once a day for 3 days. *In vitro* experiments utilized a single 6-Gy dose of radiation. We observed SOD2 expression of HT-29 and CCD 841 CoN cells stably transfected with pLVX-C9BC-SOD2-T2A-AcGFP1 after the 6-Gy doses of radiation. The SOD2 expression was examined by use of the Western blot method 48 hours after radiation treatment. Results showed that the SOD2 expression of HT-29 stably transfected cells were significantly higher after radiation treatment compared with other groups. The SOD2 expression of CCD 841 CoN stably transfected cells was significantly higher than other groups 48 hours after radiation treatment (Figure 2A–2D).

### The effects of radiation-induced SOD2 overexpression on cell proliferation

A Cell Counting Kit-8 was used to detect the alteration in cell proliferation in different experiment groups. The HT-29 cancer cell proliferation in the SOD2 + Radiation (SOD2 + R) group was inhibited markedly with a nadir at 72 hours after radiation (Figure 2E). However, the proliferation of the CCD 841 CoN normal cell in the SOD2 + R group showed improvement. with a maximum point at 72 hours after radiation treatment (Figure 2F).

### SOD2 overexpression reduces plating efficiency after radiation treatment

**Figure 3:**
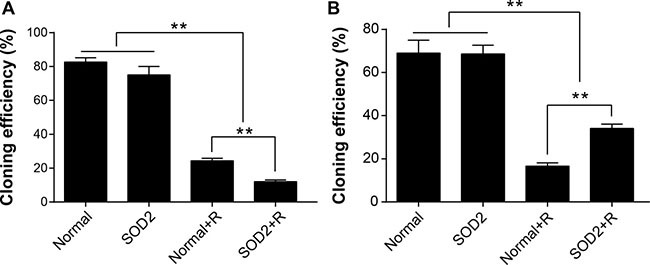
SOD2 overexpression induced by radiation-inhibited clone-forming of HT-29 cancer cells while promoting clone-forming of CCD 841 CoN normal cells (**A**) Comparison of cloning efficiency of HT-29 cells receiving different treatments. (**B**) Comparison of cloning efficiency of CCD 841 CoN cells receiving different treatments. ^*^P < 0.01. Data are shown as the means ± SD from three independent experiments. The group definition is the same as Figure 1.

Clone formation assay was utilized to evaluate the capacity of proliferation and growth of individual cells. Experimental results showed that 2 weeks after 6-Gy radiation treatment, the plating efficiency of HT-29 SOD2 stably transfected cells was significantly lower than Normal + Radiation (Normal + R) and other groups. The plating efficiency of CCD 841 CoN SOD2 stably transfected cells was significantly reduced after radiation treatment but relatively higher than Normal + R cells (Figure 3A, 3B).

### The comparison of cell apoptosis between different treatment groups

**Figure 4:**
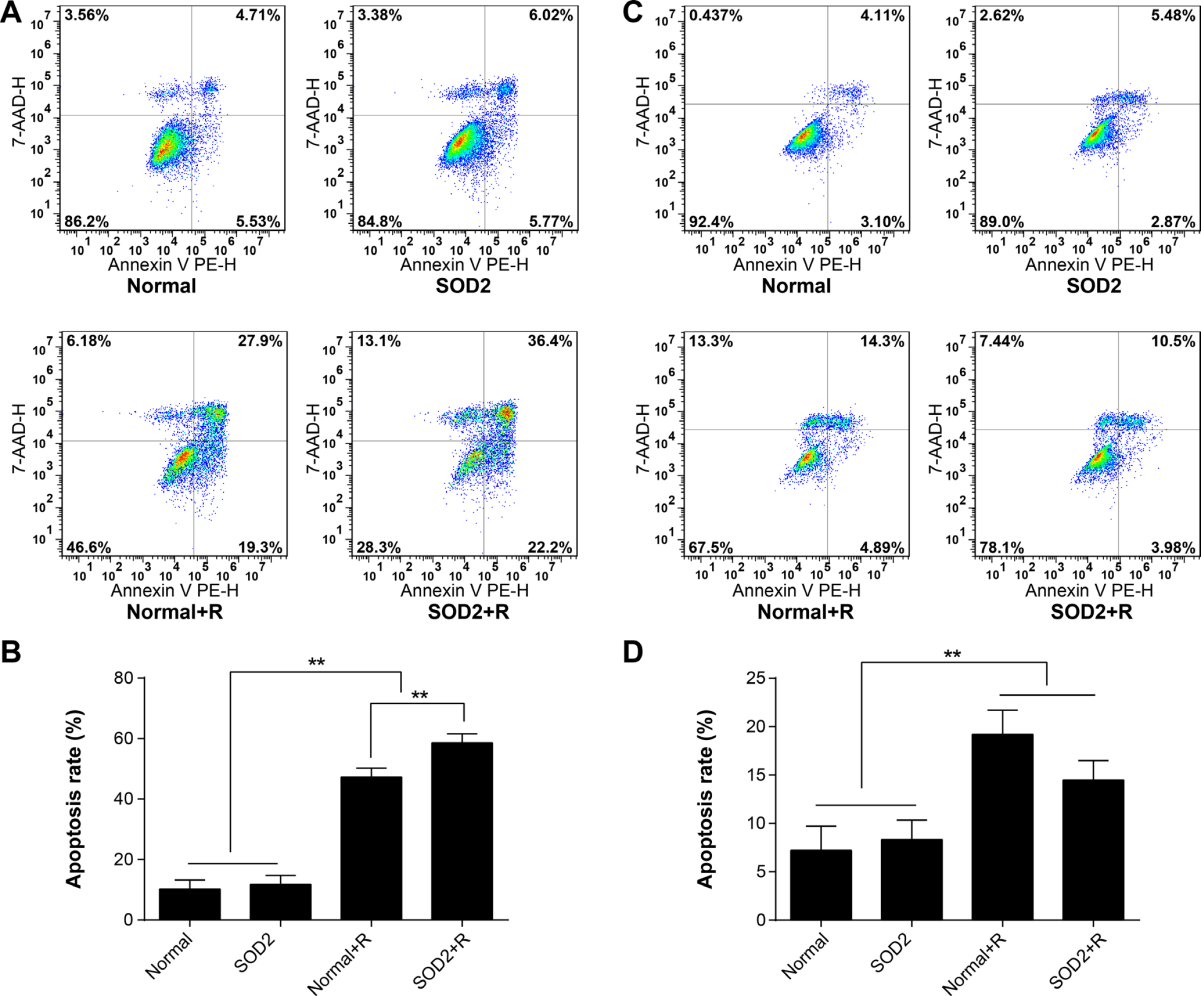
Apoptosis ratio of HT-29 and CCD 841 CoN cells receiving different treatments Apoptosis was determined by flow cytometry. (**A**) Representative diagrams of the distribution of stained HT-29 cells of different groups. (**B**) Comparisons of apoptosis ratio of HT-29 cells. (**C**) Representative diagrams of the distribution of stained CCD 841 CoN cells of different groups. (**D**) Comparisons of apoptosis ratio of CCD 841 CoN cells. ^*^P < 0.01. Data are shown as the means ± SD from three independent experiments. The group definition is the same as Figure 1.

After a 6-Gy gamma ray radiation treatment, the cell apoptosis rate of the HT-29 SOD2 + R group was significantly increased compared with the other groups (Figure 4A, 4B). The cell apoptosis rate of CCD 841 CoN SOD2 stably transfected cells showed a clear trend of decreasing compared with the Normal + R group (Figure 4C, 4D). The findings showed that SOD2 overexpression could promote cell apoptosis in colorectal cancer while inhibiting cell apoptosis in normal colon epithelial cell.

### Tumor growth curve and tumor growth inhibition rate in tumor-bearing athymic mice

**Figure 5:**
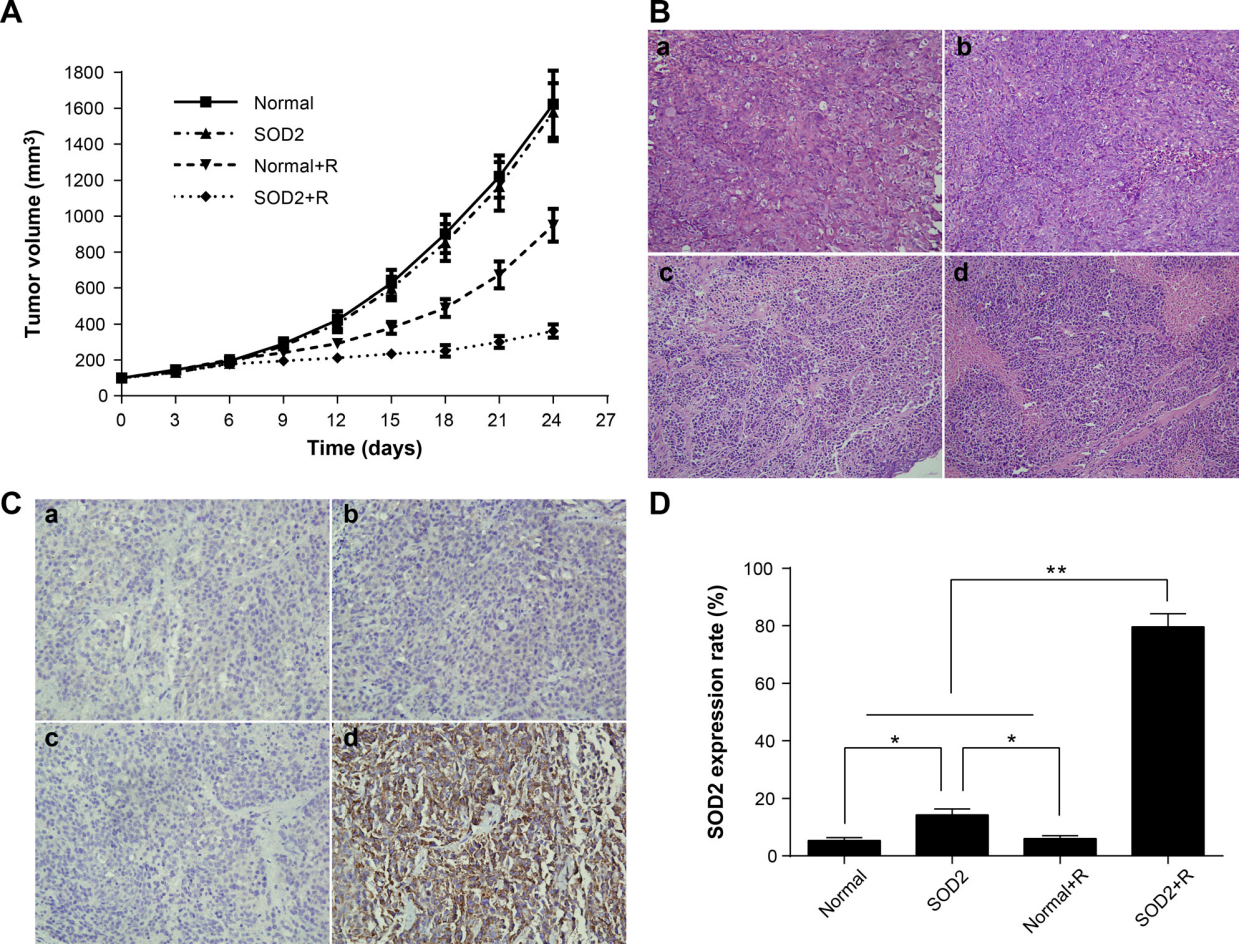
Radiation-induced overexpression of SOD2 inhibited the growth of xenografts (**A**) Tumor growth curves of different groups of tumor-bearing nude mice after treatment. (**B**) H&E staining of tissue paraffin sections from different groups of mice. a: Normal; b: SOD2; c: Normal + R; d: SOD2 + R. (**C**) Detection of SOD2 expression in tumor tissues of different groups by immunohistochemistry. a: Normal; b: SOD2; c: Normal + R; d: SOD2 + R. (**D**) Comparisons of SOD2 expression levels of different treatment groups. *P < 0.05; ^*^P < 0.01. Data are shown as the means ± SD from three independent experiments. Normal: xenografts without treatment. SOD2: xenografts receiving therapeutic lentivirus injection. Normal + R: xenografts receiving radiation treatment. SOD2 + R: xenografts receiving therapeutic lentivirus injection and radiation treatment.

Tumor volume at different time points is shown in Figure 5A. The experimental results showed that intratumoral injection of SOD2 overexpressing lentivirus can effectively inhibit tumor growth compared with other experimental treatments. The tumor inhibition rate is shown in Table 1. The results demonstrated that the tumor inhibitory effect of the SOD2 + R group was the most effective among all treatment groups. The findings indicate that SOD2 overexpression combined with radiation treatment can effectively inhibit the growth of HT-29 cancer cells *in vivo*.

**Table 1:** Tumor volume and tumor growth inhibition rate in each group after 24 days

	Normal	SOD2	Normal + R	SOD2 + R
Tumor volume (mm^3^)	1623.2 ± 186.7	1578.4 ± 161.5	949.7 ± 91.3	361.9 ± 37.0
Inhibition rate (%)	–	2.78	40.17	61.89

### Tumor pathological changes among different treatment groups *in vivo*

The tumor-bearing nude mice were sacrificed 2 weeks after radiation treatment. The tumor mass was resected, fixed in 4% formaldehyde, and examined by routine histopathological methods. The tumor cells showed a series of features under light microscope, including irregular shape, arrangement disorder, karyoplasmic ratio imbalance, nuclear hyperchromatism, and a pathological karyokinesis phase. Focal necrosis can be seen in the central region of some tumor masses. The state of cell growth was poor and more necrotic foci were found in tumor tissues of the SOD2 + R group compared with other treatment groups (Figure 5B).

### SOD2 expression in different treatment groups by immunohistochemistry *in vivo*

The results of tumor SOD2 immunohistochemistry staining are shown in Figure 5C and 5D. Positive staining of SOD2 is seen in the cytoplasm of colorectal cancer cells, in which brown-yellow particles are dispersed. The expression of SOD2 in tumor cells was significantly increased in the SOD2 + R group compared with other groups. The chimeric promoter can effectively be induced by radiation treatment, and then activate the downstream gene SOD2 expression *in vivo*.

### The variations of apoptosis *in situ* were evaluated by TUNEL *in vivo*

**Figure 6:**
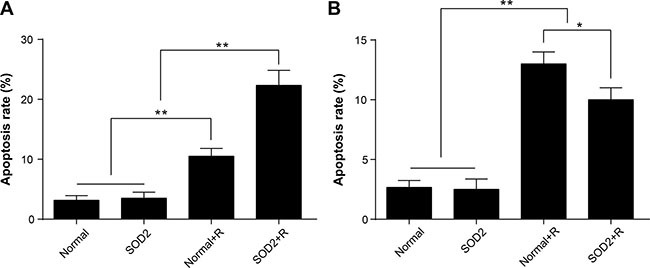
Radiation-induced overexpression of SOD2 elevated apoptosis of tumor tissues while decreasing apoptosis of peritumoral skin tissues Apoptosis *in situ* was detected by use of the TUNEL method. (**A**) Comparisons of apoptosis rate of tumor tissue. (**B**) Comparisons of apoptosis rate of skin tissue. *P < 0.05; ^*^P < 0.01. Data are shown as the means ± SD from three independent experiments. Normal: xenografts and peritumoral skin tissues without treatment. SOD2: xenografts and peritumoral skin tissues receiving therapeutic lentivirus injection. Normal + R: xenografts and peritumoral skin tissues receiving radiation treatment. SOD2 + R: xenografts and peritumoral skin tissues receiving therapeutic lentivirus injection and radiation treatment.

In the xenograft tissues, the proportion of apoptosis *in situ* was significantly higher in the SOD2 + R group than in the Normal + R group and other treatment groups (Figure 6A). In the peritumoral skin tissue, the apoptosis rate in the Normal group and the SOD2 group was comparatively low. The apoptosis rate was significantly increased in the Normal + R group. The apoptosis rate showed a clear decreased trend in the SOD2 + R group compared with the Normal + R group (Figure 6B). The above results suggested that radiation-induced SOD2 expression has a protective effect in normal skin tissue within the radiation field.

## DISCUSSION

Colorectal cancer is one of the most common malignant tumors in the gastrointestinal tract. The incidence and mortality of colorectal cancer is gradually increasing with the change in diet and lifestyle. The present comprehensive treatment of colorectal cancer includes surgery, radiotherapy, chemotherapy, and immunotherapy [15]. Radiotherapy is an important treatment in colorectal cancer [16]. However, colorectal cancer is relatively insensitive to conventional radiotherapy [17]. Increasing the dose of radiation causes radiation injury to normal tissue in the radiation field. Improving the radiosensitivity of tumor cells while reducing the radiation injury to normal tissue is an urgent problem to be solved. Our present study showed that radiation-induced SOD2 gene expression enhances the radiosensitivity of colorectal cancer cells while demonstrating radioprotective effects on colon epithelial cells and skin tissue.

SOD2 regulates various cellular processes, such as energy metabolism, cell cycle, cell proliferation, and apoptosis [18, 19]. SOD2 can reduce the toxic effects of radiation on cells and improve cell resistance to radiation. Thus, SOD2 is also considered to be an effective radiation protection agent [20, 21].

Studies have shown that SOD2 can act as a tumor suppressor, which inhibits tumor proliferation, invasion and metastasis, as well as promote apoptosis of tumor cells [22–25]. Previous studies also showed that overexpression of SOD2 in tumor cells can enhance radiosensitivity of tumor cells rather than act as a protector [12, 26, 27]. The relevant mechanism, which is unknown at present, may be the different redox state in tumor cells versus the normal cells [11, 26].

Overexpression of SOD2 within the tumor cells is believed to produce excessive hydrogen peroxide that disrupts redox balance and aggravates the oxidative damage, in turn, suppressing tumor cell proliferation and promoting apoptosis of tumor cells. Thus, promotion of SOD2 expression in both the tumors and the surrounding normal tissue within the radiation field could increase the radiosensitivity of tumors while reducing the radiation damage in normal tissue, which could effectively solve the current problems encountered in radiotherapy, such as increasing tumor radiosensitivity but aggravating normal-tissue damage.

Studies showed that the Egr-1 promoter containing CArG regulatory sequences can control the expression of downstream genes spatially and temporally [28–33]. Our previous studies found that a chimeric promoter containing six tandem CArG boxes can sense radiation treatment and activate the expression of the target gene P53 [34]. Therefore, a certain number of tandem CArG boxes can be used as a radiosensitive regulatory element to regulate the expression of the therapeutic gene [35]. A chimeric promoter composed of nine tandem CArG boxes coupled with the CMV basic promoter has better radiosensitivity and lower normal background than the wild-type Egr-1 promoter [14, 36]. Based on the preliminary work, we used the lentiviral vector pLVX-AcGFP1-N1 as backbone vector, replacing the original CMV promoter with a chimeric C_9_BC promoter containing nine tandem CArG boxes coupled with the CMV basic promoter and constructed the radiation-inducible reporter vector pLVX-C_9_BC-AcGFP1-N1 and therapeutic vector pLVX-C_9_BC-SOD2-T2A-AcGFP1.

We investigated the effects of radiation-induced overexpression of SOD2 both in human colorectal cancer cell HT-29 and in normal colorectal cell line CCD 841 CoN. *In vitro* experiments showed that radiotherapy combined with SOD2 overexpression can effectively inhibit the proliferation and growth of tumor cells and promote tumor cell apoptosis, while decreasing the apoptosis rate of normal cells after irradiation. The effects of SOD2 overexpression induced by radiotherapy was further investigated *in vivo* by use of the HT-29 xenograft model in nude mice. Radiotherapy combined with SOD2 overexpression in tumor xenografts can inhibit growth of tumor xenografts and increase the apoptosis rate of tumor cells. SOD2 overexpression in skin tissue adjacent to the tumor xenograft reduces the apoptosis rate of skin cells after irradiation. Both *in vitro* and *in vivo* studies have shown that overexpression of SOD2 combined with radiotherapy can effectively increase the radiation sensitivity of colorectal cancer cells while having radioprotective effects on colon epithelial cells and skin tissue.

In the present work, the advantages of radiation-induced SOD2 overexpression in cancer therapy are as follows. First, by controlling the radiation dose and time, target gene expression can be confined to the limited period after irradiation and be limited to the field of radiation to avoid potential side effects outside the range of radiation. Second, the effects of SOD2 overexpression on tumor cells are different from those on normal cells in tumor radiotherapy. SOD2 overexpression has “double effects,” namely, sensitizing tumor cells while protecting normal cells during radiation therapy. Third, the chimeric promoter constructed in this study is adjustable, and the expression level of SOD2 can be regulated by adjusting the radiation dose.

The present study showed that the newly constructed vector can induce the expression of target genes by radiation in a spatio-temporally confined pattern. Moreover, radiation-induced SOD2 overexpression improves HT-29 tumor cell radiosensitivity while exerting radioprotection effects on normal CCD 841 CoN cells. These findings should provide an alternative pathway for clinical application of radioactivated gene therapy.

## MATERIALS AND METHODS

### Cell lines and cell culture

The human colorectal adenocarcinoma cell line HT-29 and normal colon epithelial cell line CCD 841 CoN were obtained from the American Tissue Culture Collection (ATCC; Manassas, VA, USA) maintained in Dulbeccos Modified Eagle Medium (HyClone, Logan, Utah, USA) supplemented with 10% fetal bovine serum (Gibco, Grand Island, NY, USA) and 1% penicillin-streptomycin (Sangon Biotech, Shanghai, China), and incubated in a humidified incubator at 37°C with 5% CO_2_ and 95% air. For experimental treatment, cells were randomly divided into four groups: the normal group (cells without treatment), the SOD2 group (cells stably expressing SOD2, Supplementary Figure S2, Supplementary Table S1), the Normal + R group (cells receiving radiation treatment), and the SOD2 + R group (cells stably expressing SOD2 receiving radiation treatment). For experiments under radiation treatment, cells were exposed to 6 Gy from a radiation source (^60^CO, 0.702 Gy/min at the Radiation Center of Third Military Medical University).

### Analysis of GFP fluorescence intensity

Cells were cultured in six-well plates and collected during the logarithmic growth phase. At least 1 × 10^6^ cells per milliliter were collected and fixed with 4% paraformaldehyde for 1 hour. Samples were analyzed by flow cytometry (BD Accuri C6, USA) and the fluorescence intensity was assessed by CFlow software (BD Accuri C6). Nontransfected cells were used as negative control.

### Confocal microscopy

Cells were plated onto MatTek glass-bottomed culture dishes and allowed to adhere overnight. After treatment, cells were washed with PBS and fixed with 4% paraformaldehyde. After being washed gently with PBS twice, the cells were incubated with Hoechst 33342 staining solution for 5 minutes. Images were recorded using a Zeiss confocal microscope.

### Western-blotting analysis

The cells were lysed with Cell lysis buffer (Beyotime company, Nantong, Jiangsu, China) containing protease inhibitors. Proteins of the cell lysates were quantified using the BCA method (Beyotime), and the concentrations of samples in all groups were adjusted to the same level. Equal amounts of total proteins were separated on 12% SDS-polyacrylamide gel. Then the proteins were transferred onto a 0.2 μm polyvinylidene fluoride (PVDF) membrane using the wet transfer method at 90 V for 2h. The membranes were blocked and incubated with primary antibodies overnight at 4°C and secondary antibodies for 1 h at 37°C. After the membrane was washed with PBST, the proteins were detected using the enhanced chemiluminescence (ECL) method (Byotime). Finally, the targeted proteins were detected using the enhanced chemiluminescence (ECL) method (Byotime). β-actin was used as a control and obtained from Beyotime Biotechnology.

### Assays for cell apoptosis

Cells were trypsinized, washed twice with PBS, and collected by centrifugation, and 5 μL of 7-AAD staining solution were mixed well with 50 μL of binding buffer and added to 1 × 10^6^ collected cells. After incubation for 15 minutes at room temperature, 450 μL of binding buffer were added to the above solution, and 1 μL of Annexin V-PE was mixed with the above solution and incubated for 15 minutes. Samples were analyzed by a BD Accuri C6 flow cytometer.

### Assays for cell proliferation

Cell proliferation was evaluated by use of the Cell Counting Kit-8 (Beyotime). Cells were seeded in 96-well plates at a concentration of 2000 cells per 100 μL for each well. At 0, 24, 48, and 72 hours, 10 μL of CCK-8 solution were added to each well and incubated at 37°C for 30 minutes. The absorbance (450 nm) of each well was measured by use of a microplate reader (SpectraMax, USA).

### Plate colony formation experiments

Cells in the logarithmic growth phase were dissociated and centrifuged. The cell pellet was resuspended, and the cell concentration was adjusted to 1 × 10^5^/mL. Cell suspensions of 100 μL were inoculated onto 6-cm plates; after culture medium was added, the cells were placed in an incubator for conventional culturing for 2 to 3 weeks. When visible colonies appeared, the culture was terminated. The cells were washed with PBS twice, fixed in methanol for 15 minutes, stained with crystal violet for 20 minutes, washed with tap water, and air dried. Colony formation rate = (number of colonies/number of inoculated cells) × 100%.

### Tumor-xenograft model

Female BALB/c nude mice, 5 weeks old, were maintained under specific pathogen-free conditions in the Experimental Animal Center of Third Military Medical University. HT-29 cells in the logarithmic growth phase were dissociated and resuspended in serum-free medium at a concentration of 1 × 10^8^/mL, and 100 μL of resuspended cells were added to 100 μL of precooled matrigel and mixed well. The mixture was injected into either flank of the mice subcutaneously. When tumor volume in tumor-bearing mice reached 60 mm^3^, the mice were randomly divided into four groups (*n* = 6 mice per group): the normal group (xenografts without treatment), the SOD2 group (xenografts and peritumoral skin tissues receiving 1 × 10^8^ TU therapeutic lentivirus injection, once a day for 3 days), the Normal + R group (xenografts receiving 2 Gy of gamma ray radiation, once a day for 3 days), and the SOD2 + R group (xenografts and peritumoral skin tissues receiving therapeutic lentivirus injection, once a day for 3 days; 72 hours later, given radiation treatment, once a day for 3 days). All the mice were monitored by measuring tumor growth and ordinary circumstances every 2 days. The tumor volume = length × width^2^ / 2. Tumor growth inhibition rate = (1−tumor mean volume in treatment group/tumor mean volume in control group). All the animals were sacrificed 14 days after the treatment. Xenografts and peritumoral skin tissues were removed separately and fixed with 4% paraformaldehyde. Care and treatment of the animals were in accordance with the protocols of the Chongqing Medical Experimental Animal Care Commission.

### Immunohistochemical staining

Serial section slides (5 microns) were obtained from paraffin-embedded specimens, and the paraffin medium was removed. The slides were then rehydrated by being passed through descending serial alcohol dilutions. After antigen retrieval, slides were incubated in antibodies to SOD2 (1:1000, Abcam, Cambridge, MA, USA) overnight at 4°C. After washing, slides were incubated in secondary antibody (Beyotime) at 37°C for 1 hour, washed three times, visualized using DAB, rinsed in distilled water, and counterstained with hematoxylin. Finally, the slides were mounted and coverslipped with neutral balsam.

### Detection of apoptosis in xenografts

As for the detection of apoptosis *in situ* in xenografts, terminal deoxynucleotidyl transferase-mediated biotinylated UTP nick end labeling (TUNEL) assay to detect fragmented DNA *in situ* was carried out by using *In Situ* Cell Death Detection Kit, POD (Roche). The tissue section was dewaxed, rehydrated, and incubated with proteinase K working solution for 30 min at room temperature. 50 μL enzyme solution was added to 450 μL label solution and mix well to obtain 500 μL TUNEL reaction mixture, and then 50 μL of the mixture was added on each sample and incubated for 1 h at 37°C in a humidified atmosphere. 50 μL converter-POD was added on each sample and incubated for 30 min at 37°C. Subsequently, 50 μL DAB substrate was added and incubated for 10 min at room temperature, and then counterstained with hematoxylin. Finally, the slides were mounted and coverslipped with neutral balsam.

### Statistical analysis

e SD of three independent experiments. Differences between groups were compared using ANOVA. All statistical tests were two-sided, and differences were considered statistically significant whee SD of three independent experiments. Differences between groups were compared using ANOVA. All statistical tests were two-sided, and differences were considered statistically significant when *P* < 0.05.

## SUPPLEMENTARY MATERIALS FIGURES AND TABLES


