# Supplementary material for: Radiation-induced SOD2 overexpression sensitizes colorectal cancer to radiation while protecting normal tissue
Source: Oncotarget. 2016 Dec 15;8(5):7791–800. doi: 10.18632/oncotarget.13954 (PMC5352361; doi:10.18632/oncotarget.13954)
Supplement: Supplementary file 1 [file oncotarget-08-7791-s001.pdf]

Radiation-induced SOD2 overexpression sensitizes colorectal cancer to radiation while protecting normal tissue

Supplementary Materials

Construction of lentiviral vectors

The lentiviral vector pLVX-AcGFP1-N1 (Takara Biomedical Technology, Beijing, China) was used as backbone vector (Supplementary Figure S2A). The chimeric promoter C9BC fragment was synthesized by the Beijing Genomics Institute (BGI, Beijing, China). The 5′ end of the gene fragment contains restriction site Cla I (Takara, Dalian, China), and 3′ end contains Xho I (Takara). The C_9BC fragment and the backbone vector were digested by Cla I and Xho I respectively, purified by Gel Extraction Kit (Omega Bio-tek, Norcross, Georgia, USA), and linked by ligase to generate the reporter vector pLVX-C_9BC-AcGFP1-N1 (Supplementary Figure S2B).

The SOD2 cDNA sequence was achieved by RT-PCR and amplified by PCR method, with 5′ end containing restriction site Xho I and 3′ end containing BspT104 I. The SOD2 cDNA amplified products and the reporter vector pLVX-C_9BC-AcGFP1-N1 were digested by Xho I and BspT104 I respectively, purified by Gel Extraction Kit, and linked by ligase to generate the intermediate vector pLVX-C_9BC-SOD2-AcGFP1 (Supplementary Figure S2C).

Two complementary T2A single strand DNA sequences were synthesized by BGI, with 5′ end containing restriction site BspT104 I and 3′ end containing Apa I. The two complementary DNA sequences were annealed in annealing buffer. The intermediate vector pLVX-C_9BC-SOD2-AcGFP1 was digested by BspT104 I and Apa I. Subsequently, the annealed T2A DNA sequence and the purified vector restriction fragment were linked by ligase to generate the therapeutic vector pLVX-C_9BC-SOD2-T2A-AcGFP1 (Supplementary Figure S2D).

Packaging of lentivirus and infection of HT-29 cells

The three-plasmid package system (Addgene, Cambridge, Massachusetts, USA. packaging plasmid psPAX2 and envelop plasmid pMD2.G) was used for virus package manipulation in a grade 2 biosafety cabinet. 293FT cells were used, and plasmid transfection was performed with liposomes. After 48 h and 72 h, virus supernatant was collected and ultra-centrifuged at 50000 × g for 2.5 h. After the virus pellet was dissolved in 100 μL of Opti-MEM, virus titers were determined using quantitative PCR. Target cells were seeded, and the amount of virus was calculated based on the multiplicity of infection (MOI) value of the cell line. After 48 h of infection, stably transfected cell lines were screened using puromycin.

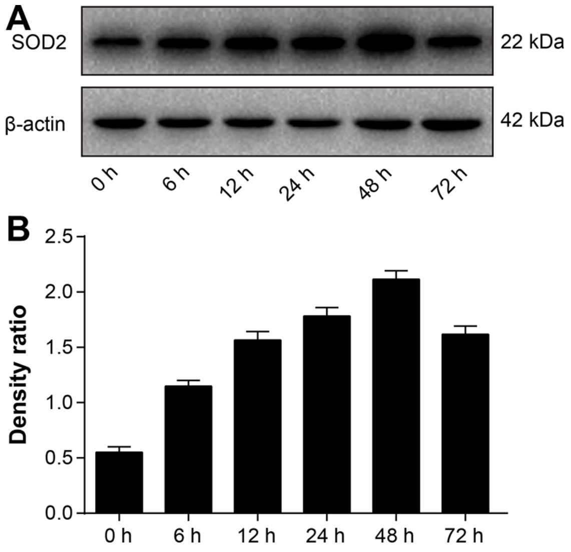

Supplementary Figure S1: Analysis of radiation-induced features of the therapeutic vector. (**A**) The SOD2 expression in HT-29 cells was assessed by Western blot. β-actin served as an internal control. (**B**) Quantification of SOD2 expression at different time after receiving 2Gy doses of radiation.

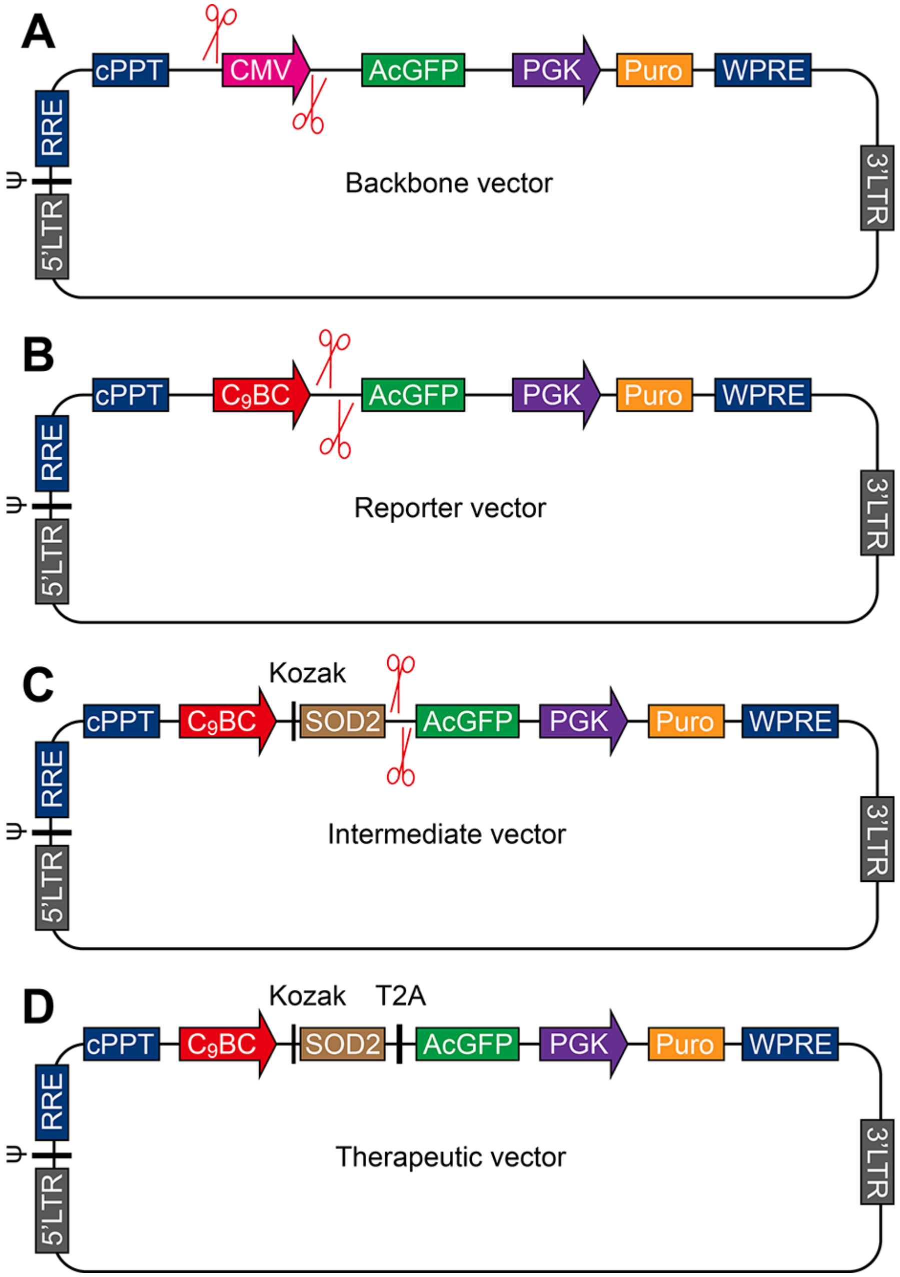

Supplementary Figure S2: The construction of lentiviral expression vectors used in this study. (**A**) the backbone vector (pLVX-AcGFP1-N1): The green fluorescent protein (GFP) is driven by human cytomegalovirus immediate early promoter (PCMV). (**B**) the reporter vector (pLVX-C9BC-AcGFP1-N1): The PCMV was replaced by C9BC chimeric promoter by digestion and connection techniques. (**C**) the intermediate vector (pLVX-C9BC-SOD2-AcGFP1): The coding sequences of SOD2 was placed downstream of C9BC promoter. (**D**) the therapeutic vector (pLVX-C9BC-SOD2-T2A-AcGFP1): The T2A sequence was inserted between the coding sequences of SOD2 and GFP and was kept in-frame with the GFP coding sequence .

Supplementary Table S1: Primers used in this study

primer	sequence	template
C_9BC amplifying F	CGACGGCCAGTCAA	C_9BC-PUC19
C_9BC amplifying R	CGCCAAAAGCTTCTC	C_9BC-PUC19
C_9BC identifying F	TTCGGGTTTATTACAGGG	pLVX-C9BC-AcGFP1-N1
C_9BC identifying R	GTGTCTTCTATGGAGGTCAA	pLVX-C9BC-AcGFP1-N1
C_9BC sequencing F	TGATAGTAGGAGGCTTGGTAGGTT	pLVX-C9BC-AcGFP1-N1
C_9BC sequencing R	CATGCCGTGGGTGATGG	pLVX-C9BC-AcGFP1-N1
SOD2 amplifying F	GCGCTCGAGGCCACCATGTTGAGCCGGGC	cDNA of SOD2
SOD2 amplifying R	CGCTTCGAACTTTTTGCAAGCCATGTATCTTTCAG	cDNA of SOD2
SOD2 identifying F	CGACCTGCCCTACGACT	pLVX-C9BC-SOD2-AcGFP1
SOD2 identifying R	GCTTCCAGCAACTCCC	pLVX-C9BC-SOD2-AcGFP1
SOD2 sequencing F	GCTGTTTTGACCTCCATAGA	pLVX-C9BC-SOD2-AcGFP1
T2A annealing F	CGAAGAGGGCAGAGGAAGTCTTCTAACAT GCGGTGACGTGGAGGAGAATCCCGGCCCTCGGGCC	–
T2A annealing R	CGAGGGCCGGGATTCTCCTCCACGTCACCGCATG TTAGAAGACTTCCTCTGCCCTCTT	–
T2A identifying F	ACCTGCCCTACGACTACG	pLVX-C9BC-SOD2-T2A-AcGFP1
T2A identifying R	AAGACTTCCTCTGCCCTC	pLVX-C9BC-SOD2-T2A-AcGFP1
T2A sequencing F	GCTGTTTTGACCTCCATAGA	pLVX-C9BC-SOD2-T2A-AcGFP1
WPRE amplifying F	CGCGTCTGGAACAATCAACC	cDNA of WPRE（realtime）
WPRE amplifying R	GTTGCGTCAGCAAACACAGT	cDNA of WPRE（realtime）
GAPDH amplifying F	AGCCTCAAGATCATCAGCAA	cDNA of GAPDH（realtime）
GAPDH amplifying F	GTCATGAGTCCTTCCACGATAC	cDNA of GAPDH（realtime）
